# Modern Medico Psychology and Psychiatry

**Published:** 1894-06-23

**Authors:** T. S. Clouston

**Affiliations:** Lecturer on Mental Diseases, Edinburgh University, and Physician Superintendent Royal Asylum, Morningside


### Modern Medico-Psychology and Psychiatry.

By T. S. Clouston, M.D., Lecturer on Mental Diseases, Edinburgh University, and Physician Superintendent Royal Asylum, Morningside.

### THE FORMS AND CLASSIFICATION OF MENTAL DISEASES.

The first scientific mental act in regard to any new subject is to observe, the second is to compare, and the third is to classify. The first real classification of mental diseases was made by Pinel, and extended by his pupil Esquirol. Its basis was the principle of assorting together similar morbid mental conditions. Little account was taken of causation, or of bodily symptoms, or of pathological changes in the structure of the brain.

*Melancholia.*—The first, and what we now know to be the basal form of insanity looked at from the mental point of view, was melancholia, or Esquirol's lypémanie. This comprised all the cases where morbid depression is the prominent and dominating mental symptom. It was the "melancholy madness" of literature, in which the joy of existence was gone; in which the primary instinct of all living beings with any mind, the desire to live and to struggle to maintain life is gone; and in which in the most pronounced cases, comprising four-fifths of the whole, there is an actual desire to die—a death-love instead of the normal universal life-love of animated nature. The reasoning powers are not so much involved as the feelings and the organic instincts. The worst cases crave for death, and try to attain it with as great an intensity as the healthy man craves to live and struggles to avoid death. The great characteristic of melancholia is mental pain, whose bodily analogue is neuralgia. Melancholia is in some degree compatible with self-control, with high effort, with intellectual acuteness, and even with genius in a few individuals. Indeed, it has often enough been the heritage of the great men of literature, of the poets, and even of some men of science and action. Mere sane melancholy and depression of spirits may run imperceptibly into melancholia. To constitute real melancholia there must be a pathological state of brain. No amount of mere depression from "temperament" or produced by real causes of distress constitutes the melancholia of the psychiatrist. They differ as the pain from a squeeze differs from the pain of neuralgia. Depression caused by losses and misfortunes is strictly physiological and natural. Nearly all other forms of mental diseases are ushered in by morbid depression of feeling, which is often enough the danger signal that should direct attention to something beginning to go wrong, like pain in bodily disease. Fortunate is the man who, when he begins to feel that it is no longer a pleasure to him to work, to eat, to sleep, to laugh, to see his friends, to live, says to himself, "There is something wrong with me, I must see to it in time." Melancholia in nine cases out of ten is preceded and accompanied by bodily symptoms of weakness, by falling off in flesh, by neuralgic pains, by want of appetite and indigestion, and by sleeplessness. It is, in fact, largely a disease from lowered brain innervation in those predisposed to mental disease. The surplus nervous energy over and above what is needed for daily use, that most valuable of all a man's qualities, the balance on the right side of the account of brain life, has been running down, the spare energy is gone in commencing melancholia. Not one of those symptoms should be neglected by a man who knows he has a hereditary tendency to mental disease, or even to any form of nervousness in his near relations. A melancholic, as the disease progresses, soon comes to have intellectual delusions founded on and tinctured by his affective pain. He thinks he has been a very bad man, that he has neglected his duties, that he has sinned against God, and done or been wrong in heart or life. Soon this passes into the delusion that others know this and that they avoid him, then that he is watched by the police or by enemies, and then his instinct of love of life leaves him, and he concludes life is no longer worth living, and he attempts self-destruction. The morbid depressed fancies are legion in different cases. Relatives think that a man in this state is too afraid of being injured or killed by others to do any harm to himself; the experienced mental doctor knows the contrary, that it is just in that state that a man thinks of and attempts self-destruction. The man may have his sense of humour left him at times, and may even have flashes of wit and gaiety when he is thinking of taking his life. The classical example of this is Cowper, despondent, delusional, suicidal, yet writing "John Gilpin," laughing over the fun of his poem, and making all the world laugh with him ever since. When confirmed and when advanced into the second stage, the melancholic often has the most fixed and terrible delusions. It is certain that one does not need to go to Hades to suffer the tortures of the damned. In an attack of melancholia often the best of men and of women, the gentlest lives, the purest spirits, suffer tortures through self-accusation in a way that no demon outside them could produce. To be very miserable you need to be very sensitive. The capacity to suffer pain is commonly equal to the gift of joy.

The bodily functions become greatly disordered, and there is apt to be most mischievous mental interpretations put upon such disorders. A man has no appetite, and therefore he takes little food; his bowels are constipated, therefore he takes strong purgatives; his skin is dry, therefore he takes exhausting Turkish baths; he has shooting pains therefore he goes to a hydropathic establishment and gets baths and "waters" and starvation diet as if he were a gouty gourmand; he can't sleep, and therefore he takes depressing sleeping drugs; he has no energy, therefore he accuses himself of "laziness," and puts on an extra spurt of work, or rushes into fatiguing travel; he feels disinclined for social intercourse, therefore at the cost of much strain he rushes into company; he feels "dead" religiously, and therefore takes to "special" church services. In every one of these things he is on the wrong tack. He does not realise that every one of these symptoms are results and not causes, the result of want of normal nerve power, and that what he wants is to restore his nerve batteries by rest, by change of scene, by life in the fresh air and change of employment which may be the truest rest, by tonics, by plenty of digestible light diet. His appetite and digestion are bad and his bowels are constipated, because the innervatian of the stomach and bowels and their nerve ganglia is deficient and needs building up, not purging. He has headaches and other pains, and is consciously disinclined to work, or is "lazy," because he is not making enough blood to circulate in his brain, and therefore he needs iron and other blood formers. His religion and his wife and his children are not comforts to him as of yore, because religion and domestic feeling needs a sound brain for its normal and comfortable manifestation, and over religious excitement only exhausts further the exhausted centres of feeling. He needs change even from home and its usually sweet routine. He is morbidly suspicious because fears are mental characters and symptoms of a weak and anæmic brain. Harmonious and soothing environments, conservation and building up of brain energy, fattening and nourishing the body, sufficient but not too much society of the right sort, implying no strain, removal from the scenes and auto-suggestions and painful feelings of the early period of the disease, are the indications of modern psychiatric experience for the rest and cure of melancholia. Self-control is often gone, and therefore in such cases control by others must be exercised. There is danger of suicide, and for this never sleeping watchfulness can alone be relied on.

